# Multi-Omics Analysis Reveals Immune Infiltration and Clinical Significance of Phosphorylation Modification Enzymes in Lung Adenocarcinoma

**DOI:** 10.3390/ijms26031066

**Published:** 2025-01-26

**Authors:** Deyu Long, Yanheng Ding, Peng Wang, Lili Wei, Ketao Ma

**Affiliations:** 1 The Key Laboratory of Xinjiang Endemic and Ethnic Diseases, Ministry of Education, Shihezi University Medical College, Shihezi 832000, China; 2 Center of Bioinformatics, College of Life Sciences, Northwest A&F University, Yangling 712100, China

**Keywords:** multi-omics, lung adenocarcinoma, tumor microenvironment, phosphorylation modification enzymes, immunotherapy

## Abstract

Protein phosphorylation is a dynamic and reversible modification involved in almost all cellular processes. Numerous investigations have shown that protein phosphorylation modification enzymes (PPMEs) that regulate protein phosphorylation play an important role in the occurrence and treatment of tumors. However, there is still a lack of effective insights into the value of PPMEs in the classification and treatment of patients with lung adenocarcinoma (LUAD). Here, four topological algorithms identified 15 hub PPMEs from a protein–protein interaction (PPI) network. This PPI network was constructed using 124 PPMEs significantly correlated with 35 cancer hallmark-related pathways. Our study illustrates that these hub PPMEs can affect the survival of patients with LUAD in the form of somatic mutation or expression perturbation. Consistency clustering based on hub PPMEs recognized two phosphorylation modification subtypes (namely cluster1 and cluster2) from LUAD. Compared with patients in cluster1, the survival prognosis of patients in cluster2 is worse. This disparity is probably attributed to the higher tumor mutation burden, the higher male proportion, and the more significant expression disturbance in patients in cluster2. Moreover, phosphorylation modification subtypes also have different characteristics in terms of immune activity, immune infiltration level, immunotherapy response, and drug sensitivity. We constructed a PSig scoring system by using a principal component analysis algorithm to estimate the level of phosphorylation modification in individual LUAD patients. Patients in the high and low PSig score groups demonstrated different characteristics in terms of survival rate, tumor mutation burden, somatic gene mutation rate, immune cell abundance, and sensitivity to immunotherapy and drug treatment. This work reveals that phosphorylation plays a non-negligible role in the tumor microenvironment and immunotherapy of LUAD. Evaluating the phosphorylation status of individual LUAD patients by the PSig score can contribute to enhancing our cognition of the tumor microenvironment and guiding the formulation of more effective personalized treatment strategies.

## 1. Introduction

Lung cancer is the most lethal type of cancer and the leading cause of cancer-related death in the world. It is one of the cancer types with the poorest 5-year survival rate among all cancer types [1,2]. Lung adenocarcinoma (LUAD) is the most prevalent histological subtype of lung cancer, responsible for approximately 40% of all lung cancer cases, and its incidence and morbidity are increasing [3,4,5]. LUAD is a complex disease with high heterogeneity located around the lung parenchyma. Its clinical manifestations are indolent, making it often locally advanced or metastatic at the time of diagnosis [5,6,7,8]. Although surgery, chemotherapy, radiotherapy, immunotherapy, and targeted therapy have made great progress in recent years, the prognosis of patients with LUAD remains unfavorable, which is largely due to the late diagnosis, high heterogeneity, and drug resistance of LUAD [9,10]. Therefore, a systematic and in-depth understanding of the molecular mechanism underlying LUAD is imperative for the development of precise treatment strategies.

Protein phosphorylation is a dynamic and reversible post-translational modification that mainly occurs on serine, threonine, and tyrosine. It is involved in almost every cellular process, such as signal transduction, cell division, gene expression regulation, and protein interaction [11,12]. Protein phosphorylation and the resulting cellular reactions generally involve a three-part system known as the protein phosphorylation modification enzyme (PPME). This system consists of protein kinases (PKs), which act as the “writers” by phosphorylating the substrate proteins; proteins with phosphoprotein binding domains (PPBDs), which function as the “readers” by recognizing the phosphorylation motifs and transmitting downstream signals; and phosphatases (PPs), which act as the “erasers” by removing phosphoprotein phosphorylation [13,14,15]. The writer–eraser–reader system assures the fidelity of the phosphorylation signals in the body. Disruptions and disorder in this system can lead to abnormal phosphorylation which has been considered to be the cause or consequence of various diseases, including cancer, cardiovascular disease, immunodeficiency, and metabolic disorders [14,16]. In addition, PKs and PPs that regulate human protein phosphorylation have been targeted in the treatment of various diseases, especially cancer. PKs represent highly valuable drug targets in the 21st century due to their frequent dysfunctions within signal transduction networks. Approximately 25% of drug development efforts in the world are dedicated to PKs, with over 250 PK inhibitors currently undergoing clinical trials, of which 37 have been approved by the United States Food and Drug Administration (US FDA) for use in humans [17,18,19]. PPs is widely expressed in the immune system and plays an important role in the cellular processes among almost all diseases. It is a key regulator of cell signal transduction and a feasible target that cannot be ignored in cancer treatment [20,21]. Therefore, a systematic investigation of the role of PPMEs in LUAD may contribute to the advancement of therapeutic strategies focused on phosphorylation.

A large number of studies have shown that the immune cell population in the tumor microenvironment (TME) performs a crucial role in cancer occurrence, progression, and patient prognosis [22]. Immune checkpoints refer to a large number of inhibitory pathways connected to the immune system, which are very important for maintaining autoimmune homeostasis and regulating the type, duration, and magnitude of immune responses [23,24]. Immune checkpoint therapy, which targets regulatory pathways in T cells to promote anti-tumor immune responses, has brought lasting clinical benefits to patients with a variety of cancer types [25,26]. Immune checkpoint inhibitors (ICIs) are a new class of anticancer drugs that are revolutionizing the treatment of many cancers. The most widely used ICIs are anti-cytotoxic T lymphocyte antigen-4 (CTLA-4), anti-programmed death ligand 1 (PD-L1), and anti-programmed death protein-1 (PD-1) antibodies [27,28,29,30,31,32]. It is worth noting that certain ICIs (such as anti-PD-1/PD-L1 and anti-CTLA-4) have been used to treat LUAD patients with specific immune manifestations. For example, PD-1 blockers/inhibitors nivolumab and pembrolizumab have been used to treat a variety of cancers, such as malignant non-small cell lung cancer, malignant head and neck squamous cell carcinoma, and malignant melanoma [33,34]. Phosphorylation plays a central role in regulating immune checkpoints, remodeling the immune microenvironment, and triggering antigen production or presentation, thus directly or indirectly affecting the efficacy of immune therapy [35]. Furthermore, many PKs have been identified as carcinogenic drivers, and their constitutive activity can lead to the abnormal phosphorylation of substrates. This abnormal phosphorylation in turn activates or inhibits signaling pathways implicated in various types of cancer. These pathways include the RTK/RAS, Wnt-β-catenin, Cell cycle, PI3K, p53, Notch, Myc, and Hippo pathways [36,37]. Importantly, abnormal oncogenic signaling pathways in cancer cells promote resistance to ICIs by modulating immune checkpoints and cancer immunosurveillance [34]. Over the past 31 years, the FDA has approved 20 small molecules and two monoclonal antibodies for the treatment of lung cancer, most of which are classified as EGFR and ALK inhibitors [38]. Therefore, a systematic exploration of the relationship between PPMEs and TME in LUAD may provide new perspectives into the classification and treatment of patients with LUAD.

In this study, we identified 15 hub PPMEs from the PPMEs enriched in hallmark-associated pathways based on four network topology algorithms. These hub PPMEs exhibit highly diverse genetic alterations and expression perturbations in normal and cancer samples. Two phosphorylation modification subtypes with different prognostic, gender, mutation burden, expression, and immune characteristics were identified based on the expression profiles of hub PPMEs employing consistency cluster analysis, implying that phosphorylation modification is an indispensable factor in the classification, prognosis, and treatment of patients with LUAD. Furthermore, we constructed a PSig scoring system to quantify the phosphorylation modification pattern in individual patients and predict the patient’s clinical response to immunotherapy and drugs. Taken together, the above results elucidate the significance of phosphorylation modification in LUAD, which can provide guidance for personalized strategies in LUAD treatment.

## 2. Results

### 2.1. Identification of Hub PPMEs in Lung Adenocarcinoma

**Figure 1:**
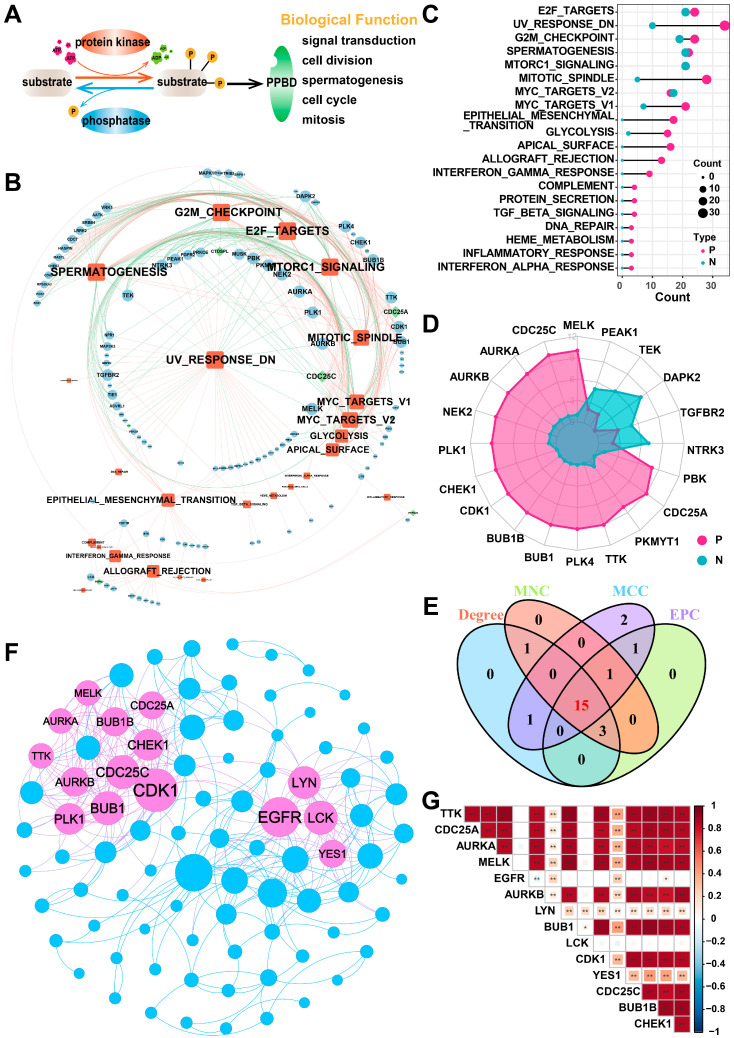
Identification of hub PPMEs in the PPI network that are significantly correlated with pathway activity. (**A**) Overview of the reversible and dynamic process of phosphorylation modification mediated by PPMEs and its underlying biological functions. (**B**) The network diagram between cancer hallmark-related pathways and PPMEs in LUAD. In the diagram, a red edge represents a significant positive correlation and a green edge represents a significant negative correlation. The size of nodes and fonts is proportional to the degree. (**C**) Number of PPMEs significantly associated with cancer hallmark-related pathways. Red indicates a positive correlation and green indicates a negative correlation. (**D**) The radar plot shows the number of cancer hallmark-related pathways that are significantly correlated with PPMEs, with red representing positive correlations and green representing negative correlations. (**E**) Venn diagram of the top 20 PPMEs in the four topological algorithms. (**F**) PPI network diagram of hub PPMEs. The size of a node is proportional to its degree. (**G**) Heatmap of correlations between hub PPMEs. * indicates a *p*-value less than 0.05, ** indicates a *p*-value less than 0.01.

In this study, we included a total of 901 PPMEs, which can dynamically and reversibly regulate the process of protein phosphorylation modification and are implicated in many potential biological functions, such as spermatogenesis, cell cycle, mitosis, and others (Figure 1A; Supplementary Materials Table S1). To investigate the potential function of PPMEs in LUAD, we calculated the Pearson correlation coefficient (PCC) between the expression of PPMEs and the activity of cancer hallmark-related pathways. The results demonstrated a significant correlation between the expression of 124 PPMEs and the activity of 35 cancer marker pathways (Figure 1B; Supplementary Materials Table S2). Among them, the activity of most cancer hallmark-related pathways were positively correlated with the expression of PPMEs. It is worth noting that the activity of 23 cancer hallmark-related pathways is significantly correlated with the expression of multiple genes, such as E2F targets, UV response DN, G2M checkpoint, spermatogenesis, and mitotic spindle (Figure 1C; Supplementary Materials Table S2). The expression of 74 genes can influence the activity of multiple cancer hallmark-related pathways, such as *MELK*, *CDC25C*, and *AURKA* (Figure 1D; Supplementary Materials Table S2). Subsequently, we constructed a PPI network based on PPMEs significantly correlated with cancer hallmark-related pathways through the STRING database. Employing four topology algorithms including Degree, Maximal Clique Centrality (MCC), Maximum Neighborhood Component (MNC), and Edge Percolated Component (EPC), fifteen PPMEs were identified as hub PPMEs, shared among the filtered PPI network (Figure 1E,F). The correlation analysis between hub PPMEs showed that except for *EGFR* and *LCK*, the expression of 13 PPMEs was significantly positively correlated, implying the unique functional mode of *EGFR* and *LCK* in LUAD (Figure 1G). *EGFR* mutations are common in LUAD, and many EGFR-targeted inhibitors (such as Dacomitinib, Erlotinib HCl, and Gefitinib) and monoclonal antibodies (such as Necitumumab) have been employed in clinical therapy [38,39]. Moreover, *MELK* exhibits high expression levels in lung cancer and is negatively correlated with the survival of LUAD patients. It can regulate the G2/M phase through the PLK1-CDC25C-CDK1 pathway, which is essential for the mitosis, programmed death, and metastasis of LUAD [40]. In summary, the above analysis has identified 15 hub PPMEs that play a crucial role in LUAD. These PPMEs have the potential to influence the survival of patients through genetic alterations or expression perturbations.

### 2.2. The Genetic Variation Landscape of Hub PPMEs in Lung Adenocarcinoma

**Figure 2:**
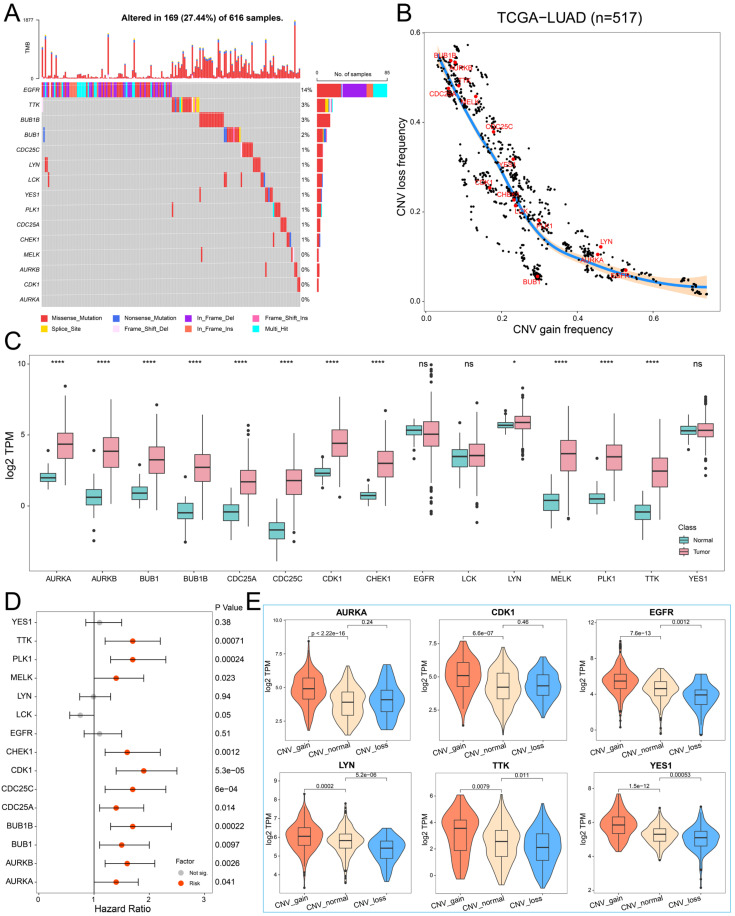
Landscape of genetic and expression perturbations of hub PPMEs in LUAD. (**A**) The mutation frequency of 15 hub PPMEs in the TCGA-LUAD cohort. The upper bar chart represents the tumor mutation burden for each patient, and the stacked bar chart on the right represents the proportion of each PPME mutation type. (**B**) CNV frequency distribution of hub PPMEs. Red dots indicate hub PPMEs and black dots indicate other PPMEs. (**C**) The expression of hub PPMEs in normal and cancer samples. The asterisk stands for the statistical *p*-value. * indicates *p*-value less than 0.05, **** indicates *p*-value less than 0.0001, and ns indicates no statistical difference between the normal and tumor groups. (**D**) Forest map of hub PPME. A red dot indicates that it is a risk factor for the overall survival of patients and a gray dot indicates that it has no effect on the overall survival of patients. (**E**) The expression of hub PPMEs in CNV groups of cancer samples. CNV_gain represents the copy number amplification of a specific gene in patients with LUAD; CNV_normal represents the copy number of a specific gene in patients with LUAD remains unchanged; and CNV_loss represents the copy number loss of a specific gene in patients with LUAD.

To investigate the genomic characteristics of hub PPMEs in LUAD, we first confirmed the somatic mutation frequency of hub PPMEs. The results showed that 27.44% of the 616 samples exhibited mutations in the hub PPMEs. Among them, the primary mutation type of hub PPMEs was the missense mutation, and *EGFR* (14%) had the highest mutation frequency (Figure 2A). The study results of CNV frequency showed that hub PPMEs have a wide range of CNV alterations. In particular, *BUB1B*, *AURKB*, *TTK*, *CDC25A*, and *MELK* had extensive copy number loss, while *LYN*, *AURKA*, and *EGFR* had widespread copy number amplification (Figure 2B). Subsequently, we explored the transcription and protein expression patterns of hub PPMEs in healthy lungs, and the results showed that *EGFR*, *LCK*, *LYN*, *YES1*, *CDK1*, and *PLK1* all had high transcription and protein expression levels, indicating that they were not or less affected by post-transcriptional regulation. It is worth noting that *TTK* was almost not expressed at the transcriptional level but had a high protein expression level, which means that the protein expression level of *TTK* is largely determined by post-transcriptional regulation (Supplementary Materials Figure S1). The expression perturbation results of hub PPMEs between normal and cancer samples revealed that hub PPMEs were significantly different between normal and cancer samples, except *EGFR*, *LCK*, and *YES1*, and they had higher expression levels in cancer samples (Figure 2C). We further used a cox regression analysis to evaluate the clinical relevance of hub PPMEs, revealing that 11 of these hub PPMEs were identified as risk factors for patient survival (Figure 2D). Interestingly, these 11 hub PPMEs have frequent protein–protein interactions and high expression correlations (Figure 1F), suggesting potential co-operation among them. In order to explore the factors of hub PPME expression disturbance in cancer samples, we calculated the correlation between CNV frequency and gene expression. The results indicate significant correlations between the expression levels of *AURKA*, *AURKB*, *BUB1*, *BUB1B*, *CDK1*, *CHEK1*, *EGFR*, *LYN*, *MELK*, *TTK*, and *YES1* with their CNV gain or loss frequencies, implying that CNV may be one of the factors contributing to hub PPME expression perturbation (Figure 2E). In conclusion, this analysis highlights the high heterogeneity of hub PPMEs in both normal and cancer samples, in terms of genetic and expression alterations. This suggests a crucial role for these hub PPMEs in the occurrence and progression of LUAD.

### 2.3. Recognition of Phosphorylation Modification Patterns Mediated by Hub PPMEs

**Figure 3:**
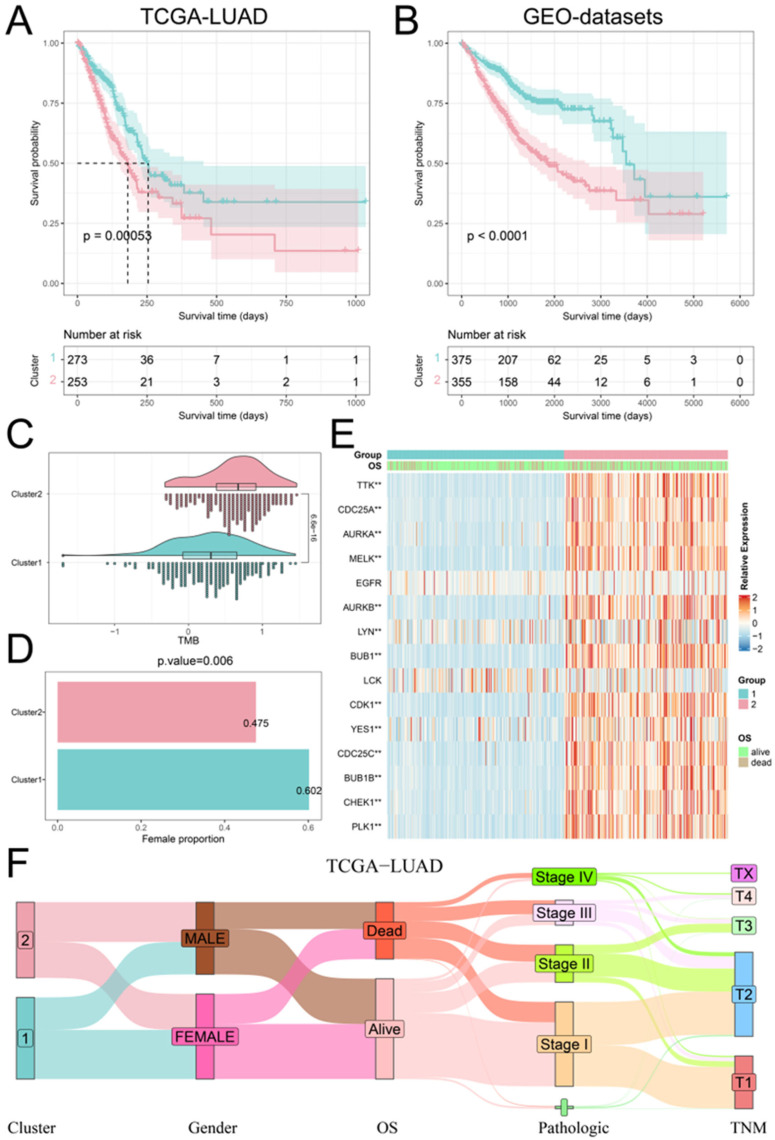
Identification and clinical characterization of phosphorylation modification patterns. (**A**) Survival analysis between the two phosphorylation modification patterns in the TCGA-LUAD cohort. The blue color refers to cluster1 and the red color refers to cluster2. (**B**) Survival analysis between the two phosphorylation modification patterns in the GEO-datasets cohort. (**C**) Tumor mutation burden between the two phosphorylation modification subtypes. (**D**) Female percentage between the two phosphorylation modification subtypes. (**E**) The expression patterns of hub PPMEs between phosphorylation modification subtypes in the TCGA-LUAD cohort. ** represents a *p*-value below 0.01. (**F**) Sankey diagram between phosphorylation modification subtypes and clinical features.

We executed a consistent cluster analysis of LUAD patients based on the expression of hub PPMEs to investigate their influence on patient classification. According to the cumulative distribution function (CDF) diagram and the consensus matrix, we determined that two categories provide the optimal segmentation for the TCGA-LUAD cohort (Supplementary Materials Figure S2A). Subsequently, the LUAD patients were divided into two phosphorylation modification subtypes: cluster1, consisting of 273 cancer samples, and cluster2, comprising 253 samples (Supplementary Materials Table S3). The survival analysis showed that patients with LUAD in cluster1 had higher overall survival compared to those in cluster2 (Figure 3A). It is worth noting that we used the same method to analyze the GEO-datasets cohort containing 730 LUAD patients. The results were similar to those of the TCGA-LUAD cohort. The optimal classification number of LUAD patients was identified as two and the patients in cluster1 had a higher overall survival rate than those in cluster2 (Figure 3B; Supplementary Materials Figure S2B; Supplementary Materials Table S4). We compared the tumor mutation burden (TMB), gender distribution, expression perturbations, and pathological stages of patients in cluster1 and cluster2 to understand the reasons for the higher overall survival rate of patients in cluster1. Compared with patients in cluster1, patients in cluster2 have a higher tumor mutation burden, which means that the high tumor mutation burden may be one of the reasons for the poor prognosis of patients (Figure 3C). Furthermore, the proportion of female patients in cluster1 was significantly higher than that in cluster2 (Figure 3D). The differential analysis of hub PPMEs between clusters demonstrated significant differences in 13 hub PPMEs between clusters 1 and 2 among the two cohorts. Notably, all 13 hub PPMEs had higher expression levels in cluster2, suggesting that the expression disorder of hub PPMEs contributes to the increased mortality rate observed in patients (Figure 3E; Supplementary Materials Figure S3). The association between phosphorylation modification subtypes and multiple clinical features in the TCGA-LUAD cohort is shown in Figure 3F. The above analysis indicates that hub PPMEs can divide LUAD patients into different phosphorylation modification subtypes. High tumor mutation burden and dysregulated expression of hub PPMEs are the potential risk factors that contribute to poor prognosis in LUAD.

### 2.4. Distinct Immune Landscapes of Phosphorylation Modification Subtypes

**Figure 4:**
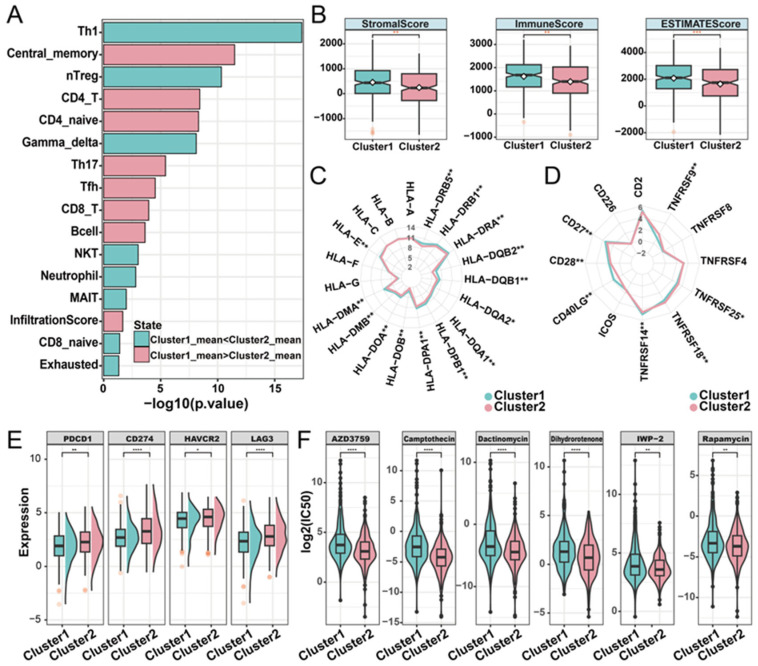
Immune characteristics of distinct phosphorylation modification subtypes. (**A**) The abundance of immune cells was significantly different between phosphorylation modification subtypes. (**B**) Stromal score, immune score, and ESTIMATE score in phosphorylation modification subtypes. ** represents *p*-value of less than 0.01. (**C**) Radar graph of median expression of major histocompatibility complex between subtypes. (**D**) Radar diagram of median expression of T-cell stimulators between subtypes. (**E**) The expression of immune checkpoint molecules in two phosphorylation modification subtypes. (**F**) The drug sensitivity of six cancer therapeutic drugs in two phosphorylation modification subtypes. * represents *p*-value of less than 0.05, ** represents *p*-value of less than 0.01, *** represents *p*-value of less than 0.001, and **** represents *p*-value of less than 0.0001.

To characterize the relationship between phosphorylation modification subtypes and immune activity, we used ImmuCellAI (Immune Cell Abundance Identifier) to investigate the tumor immune microenvironment between the two subtypes based on the expression profile data of LUAD. In both the TCGA-LUAD and the GEO-datasets cohorts, cluster1 had a higher infiltration score than cluster2. Additionally, significant differences in the abundance of nine cell types were observed. Among these cell types, Th17 (T helper 17), CD4^+^ naïve, central memory T, and CD4 T cells have high infiltration characteristics in the cluster1 subtype, while Th1, gamma delta T, nTreg (natural regulatory T cell), exhausted T, and NKT (natural killer T) cells have high infiltration characteristics in the cluster2 subtype (Figure 4A; Supplementary Materials Figure S4). Based on the expression matrix of the TCGA-LUAD cohort, the immune score, stromal score, and the ESTIMATE score (which infers tumor purity) in malignant tumor tissues were estimated by the ESTIMATE algorithm. Compared with patients in cluster2, patients in cluster1 had higher immune, stromal, and ESTIMATE scores (Figure 4B). In order to further explore the immune characteristics of phosphorylation modification subtypes, we investigated the expression differences between major histocompatibility complexes and T-cell stimulators among subtypes. Significant differences were observed in the expression patterns of the 14 major histocompatibility complexes among the phosphorylation modification subtypes, with cluster2 exhibiting high expression levels for all complexes (Figure 4C). The expression patterns of seven T-cell stimulators were also significantly different among distinct subtypes. Specifically, *TNFRSF14*, *CD40LG*, *CD27*, and *CD28* had high expression levels in cluster1, while *TNFRSF9*, *TNFRSF18*, and *TNFRSF25* had high expression levels in cluster2 (Figure 4D).

Subsequently, to investigate the potential association between phosphorylation modification subtypes and the efficacy of immunotherapy, we examined the expression levels of immune checkpoint molecules. The results showed significantly higher expression levels of *PD-1* (*PDCD1*), *PD-L1* (*CD274*), *HAVCR2*, and *LAG3* in cluster2 compared to cluster1, according to the Wilcoxon rank sum test (Figure 4E). In addition, we assessed the drug sensitivity of the two phosphorylation modification subtypes by calculating the IC50 values of cancer therapeutic drugs. This analysis revealed that AZD3759, camptothecin, dactinomycin, dihydrorotenone, IWP-2, and rapamycin had low IC50 values in both phosphorylation modification subtypes (Figure 4F). Furthermore, compared with cluster1, cluster2 has a lower IC50 value (Supplementary Materials Table S5), suggesting that patients in cluster2 exhibited higher sensitivity to drug reactions. In summary, this part of the analysis revealed the significant differences in phosphorylation modification subtypes concerning immune cell abundance, tumor purity, stromal and immune cell infiltration levels, immunotherapy response, and drug sensitivity. These findings highlight the potential value of phosphorylation modification subtypes in the classification and treatment of patients with LUAD.

### 2.5. Construction and Functional Characterization of Hub PPME Signature Scores

**Figure 5:**
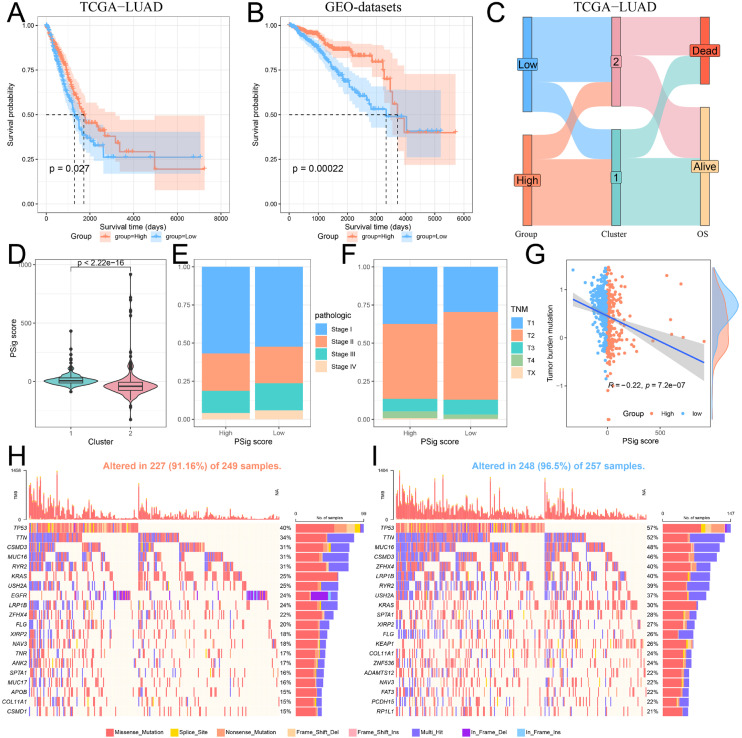
Construction and characterization of PSig scores. (**A**,**B**) Kaplan–Meier curves demonstrate survival differences between high PSig score and low PSig score groups. (**A**) The TCGA-LUAD cohort. (**B**) The GEO-datasets cohort. (**C**) The relationship between PSig score and phosphorylation modification subtypes. (**D**) The difference of PSig scores across phosphorylation modification subtypes. (**E**) The proportion of patients with different pathological stages in the high PSig score and low PSig score groups. (**F**) The percentage of patients at different stages in the high PSig score and low PSig score groups. (**G**) The correlation between PSig score and tumor mutation burden. (**H**) The waterfall diagram of the first 20 high-frequency somatic mutation genes in the high PSig score group. (**I**) The waterfall diagram of the first 20 high-frequency somatic mutation genes in the low PSig score group.

Given the importance of phosphorylation modification subtypes in the classification, prognosis, and treatment of lung adenocarcinoma patients, we established a scoring scheme called PSig score based on the expression of hub PPMEs to measure the phosphorylation modification pattern of individual LUAD patients. Subsequently, we evaluated the potential prognostic value of PSig scores. In both the TCGA-LUAD and the GEO-datasets cohorts, patients in the high PSig score group had a significantly higher survival advantage than those in the low PSig score group (Figure 5A,B; Supplementary Materials Table S6). The relationship between PSig scores and phosphorylation modification subtypes in the TCGA-LUAD cohort is presented in Figure 5C. Notably, compared with cluster2, cluster1 has a significantly higher PSig score, implying that PSig score is positively correlated with the LUAD overall survival rate (Figure 5C,D). Furthermore, the high PSig score group exhibited a higher proportion of patients with early pathological stages compared to the low PSig score group (Figure 5E,F), suggesting that the PSig score serves as a prognostic factor for LUAD. In order to evaluate the universality of PSig scores, we examined the expression of hub PPMEs in four datasets from different sequencing platforms. The results showed that most hub PPMEs were lower in the high PSig score group than in the low PSig score group (Supplementary Materials Figure S5). It is worth noting that in the GSE68465 cohort with clinical sample information, patients in the high PSig score group had a significantly higher survival advantage than those in the low PSig score group (Supplementary Materials Figure S6), which was consistent with the results in the TCGA-LUAD and GEO-datasets cohorts, suggesting the reliability and stability of the PSig score.

To investigate the correlation between PSig score and tumor somatic mutation, we compared the tumor mutation burden in different groups classified by the PSig score. Our findings revealed that the tumor mutation burden in the low PSig score group was significantly higher than that in the high PSig score group (Figure 5G). Noticeably, the PSig score was significantly negatively correlated with the tumor mutation burden. Subsequently, we used the waterfall diagram to visualize the top 20 high-frequency mutant genes in the high PSig score group and the low PSig score group. Interestingly, patients in the low PSig score group showed a higher overall gene mutation frequency than those in the high PSig score group. It is worth noting that some genes showed higher mutation frequency in the high PSig score group (Figure 5H,I). For example, the mutation frequency of *EGFR* in the high PSig score group was 24%, while the frequency in the low PSig score group was only 3.5%, hinting that the PSig score is strongly related to *EGFR* mutation. This part of the analysis constructed the PSig scoring system and explored its associations with the clinical prognosis, pathological stage, and somatic mutation patterns in patients with LUAD.

### 2.6. The Role of PSig Scores in Predicting Immunotherapy and Chemotherapeutic Efficacy

**Figure 6:**
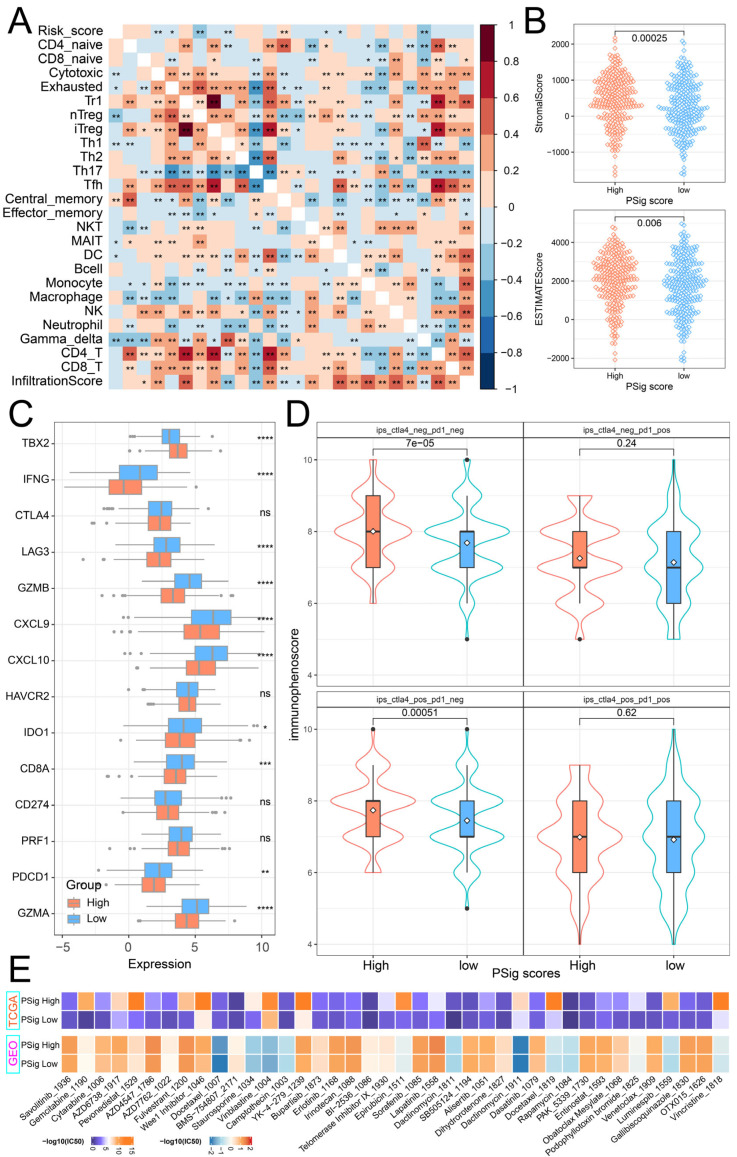
Clinical relevance of PSig scores. (**A**) The correlation heatmap between the PSig score and immune cell abundance. Red represents a positive correlation; blue represents a negative correlation. * represents a *p*-value below 0.05 and ** represents a *p*-value below 0.01. (**B**) The stromal score and the ESTIMATE score between the high PSig score group and the low PSig score group. The Wilcoxon rank sum test was used to test whether the difference between groups was significant. (**C**) The expression levels of 14 immune checkpoint-related genes in the high PSig score group and low PSig score group. The Wilcoxon rank sum test was used to test the statistical difference between the groups. * represents a *p*-value of less than 0.05, ** represents a *p*-value of less than 0.01, *** represents a *p*-value of less than 0.001, **** represents a *p*-value of less than 0.0001, and ns represents no significant difference. (**D**) The distribution map of immunophenoscore in the high PSig score group and the low PSig score group. (**E**) IC50 distribution heatmaps of significantly different drugs in the high PSig score group and the low PSig score group.

To explore the relationship between PSig score and tumor immunity, we calculated the Pearson correlation coefficient to evaluate the association between PSig score and the abundance of 24 immune cell types. The analysis revealed a significant correlation between PSig score and seven T-cell subtypes, as well as DC cells. Moreover, various other immune cell types frequently exhibited significant correlations. (Figure 6A). Through the ESTIMATE algorithm, we discovered that the stromal cell infiltration level and estimate score in the high PSig score group were significantly higher than those in the low PSig score group. However, there was no significant difference in the level of immune cell infiltration (Figure 6B). Immune checkpoint inhibitor therapy is one of the emerging immunotherapy treatments widely used in clinical practice [41]. To investigate the potential of employing PPME-focused ICI therapy in LUAD, we compared the expression differences of 14 immune checkpoint-related genes in the PSig score groups. The results demonstrate that there were significant differences in 10 immune checkpoint-related genes between the two PSig score groups. Interestingly, all nine of these genes, except for TBX2, were highly expressed in the low PSig score group (Figure 6C). Moreover, the immunophenoscore (IPS) is one of the newly discovered factors that can be used to evaluate a patient’s response to immunotherapy [42,43]. Compared with patients in the low PSig score group, patients in the high PSig score group showed significantly higher IPSs, implying clinical benefits from anti-PD-1/CTLA4 immunotherapy (Figure 6D). To assess the reaction of chemotherapy in different groups classified by the PSig score, we compared the IC50 values in different groups. In both the TCGA-LUAD and GEO cohorts, a total of 89 drugs exhibited significant differences between the high PSig score group and the low PSig score group. Among them, the IC50 values of BMS-754807, Doramapimod, SB216763, and SB505124 in the high PSig score group were lower, indicating that the high PSig score group had higher sensitivity to these four drugs. Conversely, the other 85 drugs had lower IC50 values in the low PSig score group, suggesting that the low PSig score group had higher drug sensitivity to these 85 drugs (Figure 6E; Supplementary Materials Table S7). In summary, the above analysis shows that the PSig score can be used as a key factor in predicting the treatment and prognosis of patients with LUAD.

## 3. Discussion

Protein phosphorylation occurs primarily in the cytoplasm and nucleus and is the most prevalent and studied post-translational modification. It is a basic, universal, and important mechanism regulating protein activity and function, and is involved in virtually all biological processes of cells [12,16]. PPMEs are essential for maintaining the homeostasis of the phosphorylation system. Dysregulation of the phosphorylation system is tightly associated with various human diseases such as cancer and immunodeficiency [14]. In addition, a large number of studies have shown that phosphorylation is closely related to tumor immunity, and some immune drugs related to phosphorylation have been developed and utilized, such as osimertinib, MYCi975, Simvastatin, and nintedanib [44,45,46,47,48]. PKs play an important role in regulating immune checkpoints in tumor and immune cells and reshaping the TME by invoking innate immune responses and the generation and presentation of neoantigens. The *EGFR* inhibitor gefitinib instabilises PD-L1 by activating glycogen synthase kinase 3β (*GSK3β*) and enhances the therapeutic effect of *PD-1* blockade in homozygous mouse models [35,49]. Therefore, a comprehensive elucidation of the function of PPMEs in the classification and characteristics of LUAD can potentially advance the development of personalized treatment methods and strategies for patients with LUAD.

Here, we revealed a significant association between 124 PPMEs and 35 cancer hallmark-related pathways. Additionally, we identified 15 hub PPMEs from the corresponding PPI network by four topological algorithms. The hub PPMEs are divided into two parts in the PPI network. Among them, *EGFR*, *LCK*, *LYN*, and *YES1* are closely connected in the network, but the correlation between them is low or irrelevant, suggesting that they may be acting in a genetic alternation manner, while the remaining 11 hub PPMEs are closely connected in the network and have high correlation, suggesting that they may be functioning in an expression disturbance manner. In LUAD, *EGFR* is one of the most frequently mutated oncogenic driver genes and is the target in the majority of drugs [50,51]. Studies have shown that the amplification of *YES1* is a targetable and recurrent mechanism of resistance to EGFR inhibition in EGFR-mutant lung cancer [52]. Subsequently, we revealed the genetic changes and expression disturbances of hub PPMEs in LUAD. *EGFR* has the highest mutation frequency, and there is a wide range of copy number amplifications/losses of hub PPMEs. Compared with normal samples, 12 hub PPMEs have higher expression levels in LUAD samples. Among these hub PPMEs, except for *LYN*, the activation of other hub PPMEs is a risk factor for patient survival. Our results are consistent with many previous studies. For example, the overexpression of *BUB1* and *BUB1B* promotes tumor cell proliferation and metastasis by interfering with spindle assembly checkpoints in mitosis, while the knockdown of *BUB1B* inhibits cell proliferation, invasion, and migration in vitro [53,54]. The overexpression of *PLK1* can induce epithelial-mesenchymal metastasis. In LUAD, PLK1-mediated phosphorylation of vimentin activates the TGF-β signaling pathway, contributing to metastasis and immune evasion via the expression of *PD-L1* [55,56]. The overexpression of *AURKA* and *AURKB* can enhance primary tumor growth in mouse tumor models, and their direct phosphorylation can disrupt the stability of the tumor suppressor p53 [57]. These previous studies suggest the reliability of our selected hub PPMEs.

Subsequently, we identified two phosphorylation clusters in LUAD patients with different characteristics, as determined by the expression profile of hub PPMEs. Compared with patients in cluster1, patients in cluster2 had significantly lower overall survival rates. This difference in survival may be attributed to a lower proportion of female patients, a higher tumor mutation burden, and a high expression disturbance of hub PPMEs in cluster2. In addition, the immune microenvironment is an important part of the TME, including immune cells such as lymphocytes, dendritic cells, monocytes, macrophages, and immune checkpoints such as *PD-1*, *PD-L1*, and *CTLA-4*, many of which have been considered as potential markers in clinical practice [58,59]. Our results revealed that there were significant differences in the abundance of nine immune cells, tumor purity, matrix, and immune cell infiltration levels between the two phosphorylated clusters, hinting at distinct responses to immunotherapy. Subsequently, we found that immune checkpoints *PD-1*, *PD-L1*, *HAVCR2*, and *LAG3* had higher expression levels in cluster2 than in cluster1. Additionally, patients in cluster2 exhibited greater sensitivity to drug response compared to those in cluster1. The results of this section indicate that the phosphorylation modification cluster is a valuable classification method in LUAD.

Finally, we constructed the PSig scoring system to evaluate the features of individual patient survival, somatic mutations, immune cell abundance, immune infiltration, immune checkpoints, and drug sensitivity. Our results show that the PSig score is one of the prognostic factors affecting LUAD. Compared with patients with low PSig scores, patients with high PSig scores have better survival rates and lower tumor mutation burden. Previous studies have shown that genetic alternations play an important role in the occurrence and progression of tumors, and are strongly associated with the diagnosis, treatment, and prognosis of multiple cancers [33,60,61]. It is worth noting that the mutation frequency of *EGFR* in the high PSig score group is as high as 24%, while the mutation frequency of *EGFR* in the low PSig score group is only 3.5%. EGFR-targeted therapy has opened up a new era of precision oncology. It is one of the most studied tyrosine kinase receptors involved in tumor formation in various tissues around the world [62]. Further analysis showed significant differences in immune cell abundance, immune checkpoint expression, and drug sensitivity between the two groups with distinct PSig scores, suggesting distinct patterns in immunotherapy and drug response. These findings may provide new insights into personalized immunotherapy for patients with LUAD in the future.

This work will provide a new perspective for understanding the relationship between phosphorylation status and TME in LUAD patients. However, this work still has some limitations. Although we collected and analyzed many patient samples from independent datasets, there is a lack of in-depth exploration of the diversity of the patient population. In addition, although some of the results in this study have been experimentally verified, more comprehensive clinical data and experimental support are still needed.

## 4. Materials and Methods

### 4.1. Sources and Preprocessing of Publicly Obtainable Datasets

Information on human PPMEs was sourced from the iEKPD 2.0 database [14]. A total of 901 PPMEs were curated in this study, including 523 protein kinases, 165 protein phosphatases, and 213 proteins containing phosphoprotein binding domains (Supplementary Materials Table S1). The transcriptome and proteome expression matrices of normal lungs were obtained from the Genotype–Tissue Expression (GTEx) consortium and Human Proteome Map (HPM), respectively [63,64]. The gene expression data and clinical features of LUAD patients are mainly derived from the GEO database and TCGA database. Specifically, the expression profiles of all samples in the TCGA-LUAD cohort (n = 598) were obtained via the R package TCGAbiolinks (version 2.26.0). TCGAbiolinks is a user-friendly R package that provides users with query, search, download, integrated analysis, and visualization functions, aiming to simplify the data mining and analysis process of cancer genome data stored in the Genomic Data Commons (GDC) [65,66]. The clinical information of the samples in the TCGA-LUAD cohort was derived from the GDC TCGA LUAD cohort in the UCSX XENA database. In addition, three LUAD cohorts (GSE31210 (n = 246), GSE37745 (n = 196), and GSE72094 (n = 442)) with survival information were screened out in the GEO database, and the raw CEL files and corresponding survival information were downloaded from the GEO database. The raw CEL file is background corrected and quantile normalized through the affy (version 1.76.0) package’s robust multichip average (RMA) algorithm. Then, the combat method in the sva (version 3.46.0)package is used to perform batch correction on the three sets of the GEO LUAD cohorts, and the combined dataset is referred to as the GEO-datasets (n = 730) [67]. The expression matrix and sample annotation information of GSE32863 (n = 116), GSE40791 (n = 194), and GSE68465 (n = 462) were obtained from GEO database by the R package GEOquery (version 2.66.0) [68,69,70,71]. It is worth noting that the GSE68465 cohort contains the survival information of LUAD patients. In addition, an expression matrix containing 141 never-smoker LUAD patients (accession number: GSE256091) was collected from the GEO database.

### 4.2. Identification of Hub PPMEs

Gene set variation analysis (GSVA) was performed with the R package GSVA (version 1.46.0), which offers a gene set enrichment analysis method to evaluate pathway activity variation over a sample population by non-parametric and unsupervised methods [72]. In this study, the activity of cancer hallmark-related pathways in the TCGA-LUAD cohort was estimated using GSVA, based on the expression matrix of PPMEs. The cancer hallmark-related pathways (“hallmark gene sets”) were obtained from the Molecular Signatures Database (MSigDB) through the R package msigdbr (version 1.6.0). In order to characterize the correlation between PPMEs and pathway activation or inhibition, we calculated the PCC between the expression of PPMEs and the activity of cancer hallmark-related pathways and adjusted the *p*-value by the BH (Benjamini and Hochberg) method. PPME–pathway relationship pairs were considered significantly correlated if the absolute value of the PCC was greater than 0.5 and the adjusted *p*-value was less than 0.01. Subsequently, PPMEs in significant correlated PPME–pathway pairs were extracted, and a protein–protein interaction (PPI) network was constructed through the STRING online database [73]. The PPI network was further filtered according to the interaction score, and the relationship pairs with interaction scores higher than 0.7 were retained. Then the filtered PPI network was imported into the Cytoscape (version 3.10.3) software, and the cytoHubba plug-in in Cytoscape was used to extract the top 20 critical PPMEs from the filtered PPI network by using four different algorithms, including Degree, MCC, MNC, and EPC [74]. The PPMEs jointly screened by the four algorithms were regarded as hub PPMEs.

### 4.3. Genetic Variation Analysis of Hub PPMEs

The somatic mutation data of patients in the TCGA-LUAD cohort were obtained from the GDC database through the TCGAbiolinks (version 2.26.0) package. The R package maftools (version 2.14.0) was used to visualize the somatic mutation data of hub PPMEs, and the information of patients in the TCGA-LUAD cohort was obtained via the tmb function in the maftools package [75]. Copy number variation (CNV) data in the TCGA-LUAD cohort were downloaded from the UCSX Xena database, and the frequency of CNVs in PPMEs was visualized using the R package ggplot2 (version 3.5.1). To determine if there were significant differences in hub PPMEs between normal and cancer samples in the TCGA-LUAD cohort, the Wilcoxon rank sum test was conducted. Subsequently, we calculated the Pearson correlation coefficients to assess the relationship between CNV and gene expression in hub PPMEs. Differences in expression among CNV gain, CNV loss, and normal samples in hub PPMEs were compared using the Wilcoxon rank sum test, with *p*-values below 0.05 considered significant. In addition, we performed a univariate Cox regression analysis using the R package survival to evaluate the impact of hub PPME expression on the overall survival of patients.

### 4.4. Unsupervised Clustering and Survival Analysis

The expression profiles of hub PPMEs were extracted from the TCGA-LUAD and the GEO-datasets cohorts. Consistency clustering analysis of patients was performed by the R package ConsensusClusterPlus. ConsensusClusterPlus (version 1.62.0) is an open source software that implements a consensus clustering method in R and provides confidence evaluation and project tracking capabilities [76]. The optimal number of clusters is determined based on the consensus matrix and cumulative distribution function (CDF) diagram. The Kaplan–Meier method was used to generate the survival curves of patients in different clusters of the same cohort and the significance of differences was assessed using the log-rank test. The expression differences of hub phosphorylation-modifying enzymes between clusters were compared using the Wilcoxon rank sum test and visualized using the R package ComplexHeatmap (version 2.14.0) [77]. The relationship between phosphorylation modification subtypes and clinical features was visualized by the R package ggsankey (version 0.0.99999).

### 4.5. Immune Landscape of Phosphorylation Modification Subtypes

The abundance and distribution of immune cells play a crucial role in cancer immunotherapy, especially T-cell subsets. Here, we predicted the abundance of macrophages, neutrophils, monocytes, B cells, DC cells, NK cells, and 18 T-cell subsets through ImmuCellAI (version 0.1.0) (Immune Cell Abundance Identifier) according to the expression matrix of LUAD patients. ImmuCellAI is a geneset signature-based approach that accurately assesses the abundance of 24 immune cell types and provides overall infiltration scores using gene expression datasets [78]. According to the expression data of LUAD patients, the ESTIMATE algorithm based on single-sample gene set enrichment analysis was used to predict tumor purity, and infiltrating stromal and immune cell levels in tumor tissues [79]. The Wilcoxon rank sum test was used to determine whether there were significant differences in immune cell abundance, tumor cell purity, and infiltrating stromal and immune cell levels in different clusters/subtypes. Additionally, the R package oncoPredict (version 1.2) was used to predict the half-inhibitory concentration values frequently used in cancer therapeutic drugs for each patient in the TCGA-LUAD cohort. This allowed for a comparison of their clinical drug responses across different phosphorylated modification subtypes [80]. Moreover, a series of predictive indicators such as immune checkpoints, major histocompatibility complexes, and T-cell stimulators were also employed to assess the relationship between phosphorylation modification subtypes and immunotherapy effects.

### 4.6. Construction of PSig Score

To examine the phosphorylation modification pattern of individual LUAD patients, we employed principal component analysis (PCA) to construct a scoring system called the PSig score. In detail, we performed principal component analysis based on the expression profile of hub PPMEs and selected principal component 1 and principal component 2 as signature scores. The advantage of this method is that it mainly focuses on the set’s score with the largest inverse correlation or good correlation among gene blocks, reducing the weight contribution of genes that do not track with other set members. We then defined the PSig score using a formula similar to that used in previous studies: $PSig\;score=\sum \left({PC1}_{i}{+PC2}_{i}\right)$, where i represents the expression of the hub PPME [81,82]. According to the median of the PSig score, LUAD patients were divided into high PSig score and low PSig score groups. The immunotherapy scores of patients in the TCGA-LUAD cohort were obtained from the TCIA (The Cancer Immunome Atlas) database [83], and their statistical significance in the PSig score group was compared by the Wilcoxon rank sum test. We also employed the Wilcoxon rank sum test and visualized the results using the ComplexHeatmap [77] package to explore significant differences in drug sensitivity IC50 values among the PSig score groups.

### 4.7. Statistical Analysis

All statistical analyses were conducted using the R software (version 4.2.3), with the specific R packages mentioned above. The Pearson correlation between PSig score and immune cell abundance was calculated by the R package Hmisc (version 5.1.2). Unless otherwise specified, the comparison of the difference between two groups is performed by the Wilcoxon rank sum test, with a *p*-value threshold of 0.05 indicating statistical significance.

## Data Availability

All datasets in this study are available from public online repositories, and the repository name and accession numbers are discoverable in the manuscript or Supplementary Materials.

## Supplementary Materials

The supporting information can be downloaded at: https://www.mdpi.com/article/10.3390/ijms26031066/s1.

## Abbreviations

**Abbreviation**	**Full Name**
BH	Benjamini and Hochberg
CNV	Copy number variation
CDF	Cumulative distribution function
CTLA-4	Cytotoxic T lymphocyte antigen-4
EPC	Edge percolated component
GSVA	Gene set variation analysis
GDC	Genomic data commons
GTEx	Genotype–Tissue Expression
GSK3β	Glycogen synthase kinase 3β
HPM	Human proteome map
ImmuCellAI	Immune cell abundance identifier
ICIs	Immune checkpoint inhibitors
IPS	Immunophenoscore
LUAD	Lung adenocarcinoma
MCC	Maximal clique centrality
MNC	Maximum neighborhood component
MSigDB	Molecular signatures database
NKT	Natural killer T
nTreg	Natural regulatory T cell
PCC	Pearson correlation coefficient
PPs	Phosphatases
PPBDs	Phosphoprotein binding domains
PCA	Principal component analysis
PD-L1	Programmed death ligand 1
PD-1	Programmed death protein-1
PKs	Protein kinases
PPME	Protein phosphorylation modification enzyme
PPI	Protein–protein interaction
RMA	Robust multichip average
Th17	T helper 17
TCIA	The Cancer Immunome Atlas
TME	Tumor microenvironment
TMB	Tumor mutation burden
US FDA	United States Food and Drug Administration
