# Supplementary material for: Multi-Omics Analysis Reveals Immune Infiltration and Clinical Significance of Phosphorylation Modification Enzymes in Lung Adenocarcinoma
Source: Int J Mol Sci. 2025 Jan 26;26(3):1066. doi: 10.3390/ijms26031066 (PMC11817228; doi:10.3390/ijms26031066)
Supplement: Supplementary file 1 [file ijms-26-01066-s001.zip › Supplemental_Material.pdf]

Multi-omics analysis reveals immune infiltration and clinical significance of phosphorylation modification enzymes in lung adenocarcinoma

Deyu Long[1,2], Yanheng Ding[2], Peng Wang[2], Lili Wei[1*], Ketao Ma[1*]

[1]The Key Laboratory of Xinjiang Endemic and Ethnic Diseases, Ministry of Education, Shihezi University Medical College, Shihezi, 832000, China

[2]Center of Bioinformatics, College of Life Sciences, Northwest A&F University, Yangling, Shaanxi 712100, China.

*Corresponding authors: Lili Wei, Email: wll1126@shzu.edu.cn; Ketao Ma, Email: maketao@shzu.edu.cn.

This PDF file includes:

Figure S1 to S6

Figure S1. Transcription and protein expression levels of hub PPMEs in normal lung.

Figure S2. The consistency matrix and cumulative distribution function diagram of the dataset.

Figure S3. The expression patterns of hub PPMEs between phosphorylation modification subtypes in the GEO-datasets cohort.

Figure S4. The abundance of immune cells between phosphorylation modification subtypes in the GEO-datasets cohort.

Figure S5. Expression patterns of hub PPMEs between different PSig score groups. n represents the number of LUAD patients in each cohort.

Figure S6. Kaplan-Meier curves showed the difference in survival between the high PSig and low PSig groups in the GSE68465 cohort.

Other Supplementary Materials for this manuscript include the following:

Table S1 to S7

Table S1. Information on PPMEs in humans.

Table S2. The correlation between PPMEs and cancer hallmark-related pathways.

Table S3. Consistency clustering results of patients in TCGA-LUAD cohort.

Table S4. Consistency clustering results of patients in GEO-datasets cohort.

Table S5. Drug sensitivity analysis between phosphorylation modification subtypes.

Table S6. The construction of PSig scores in TCGA-LUAD and GEO-datasets cohort.

Table S7. Drug sensitivity analysis between PSig groups.

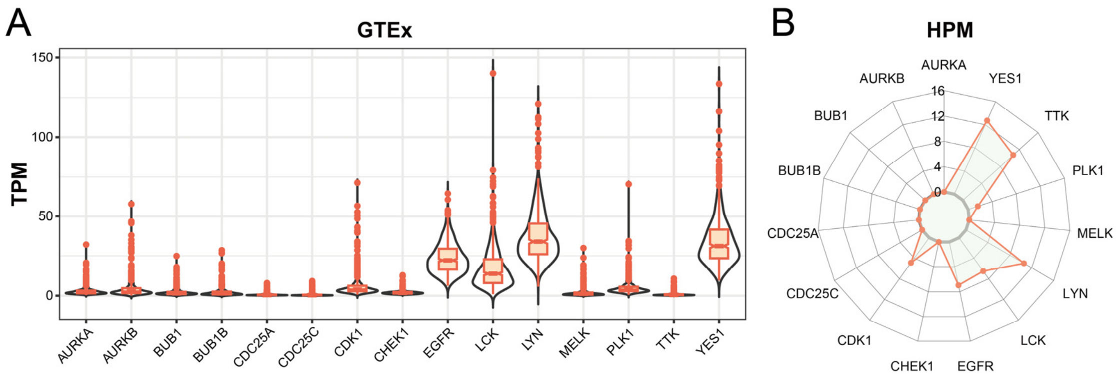

Figure S1. Transcription and protein expression levels of hub PPMEs in normal lung. (A) Transcriptional expression levels of hub PPMEs in GTEx. (B) Protein expression levels of hub PPMEs in HPM.

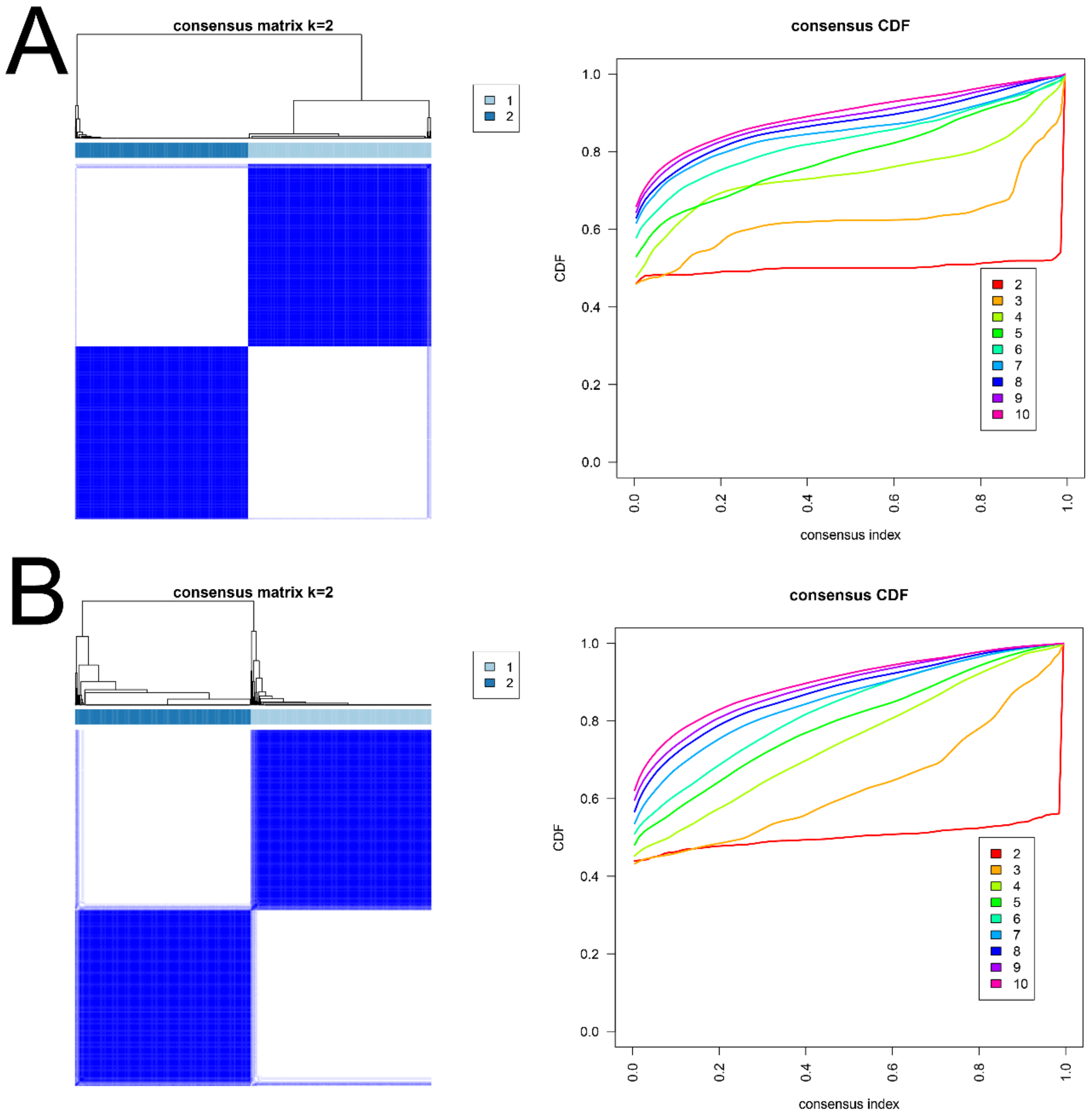

Figure S2. The consistency matrix and cumulative distribution function diagram of the

dataset. (A) TCGA-LUAD cohort; (B) GEO-datasets cohort.

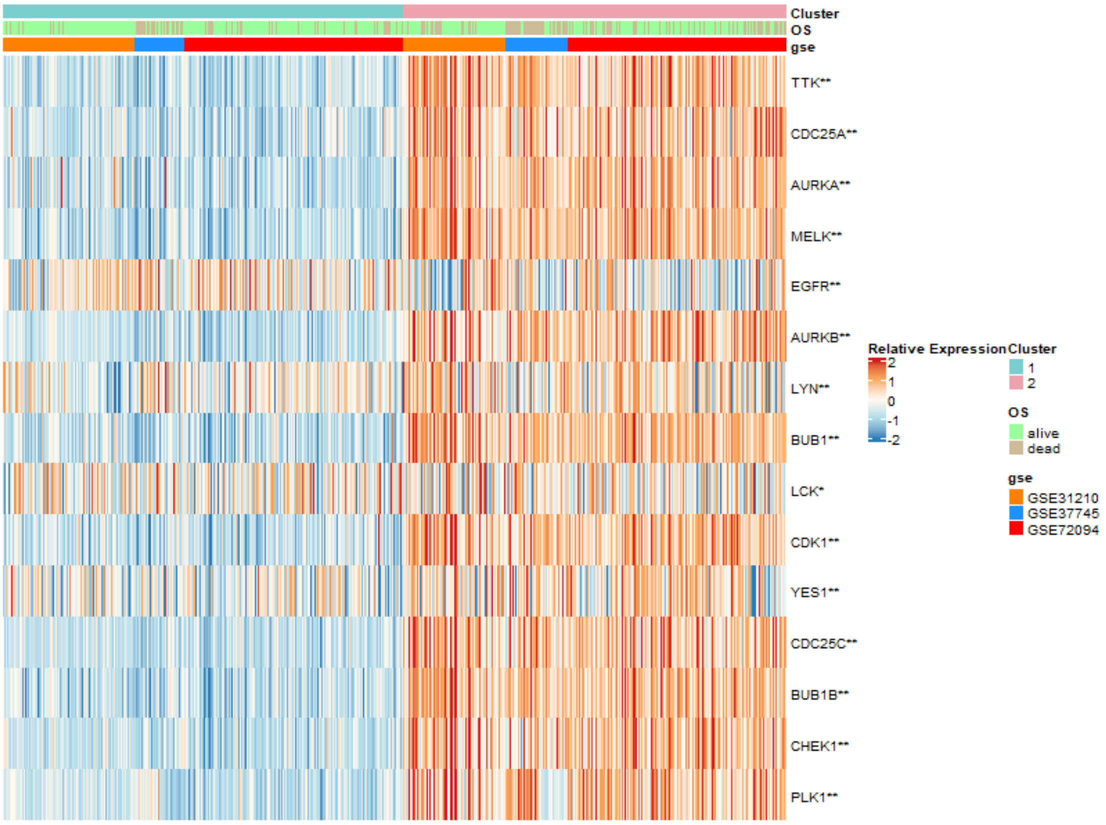

Figure S3. The expression patterns of hub PPMEs between phosphorylation modification subtypes in the GEO-datasets cohort. * represents a *p-value* below 0.01, and ** represents a *p-value* below 0.01.

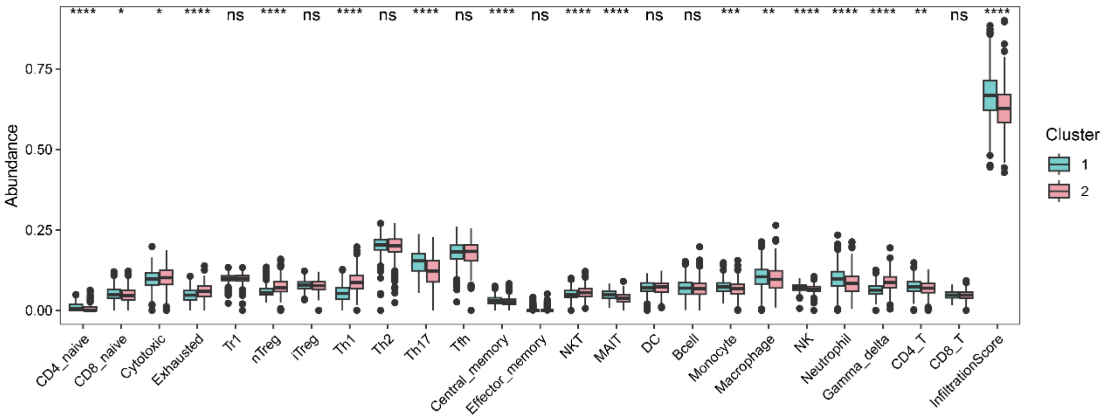

Figure S4. The abundance of immune cells between phosphorylation modification subtypes in the GEO-datasets cohort. * indicates *p-value* less than 0.05, ** indicates *p-value* less than 0.01, *** indicates p-value less than 0.001, **** indicates *p-value* less than 0.0001, and ns represents no significant difference.

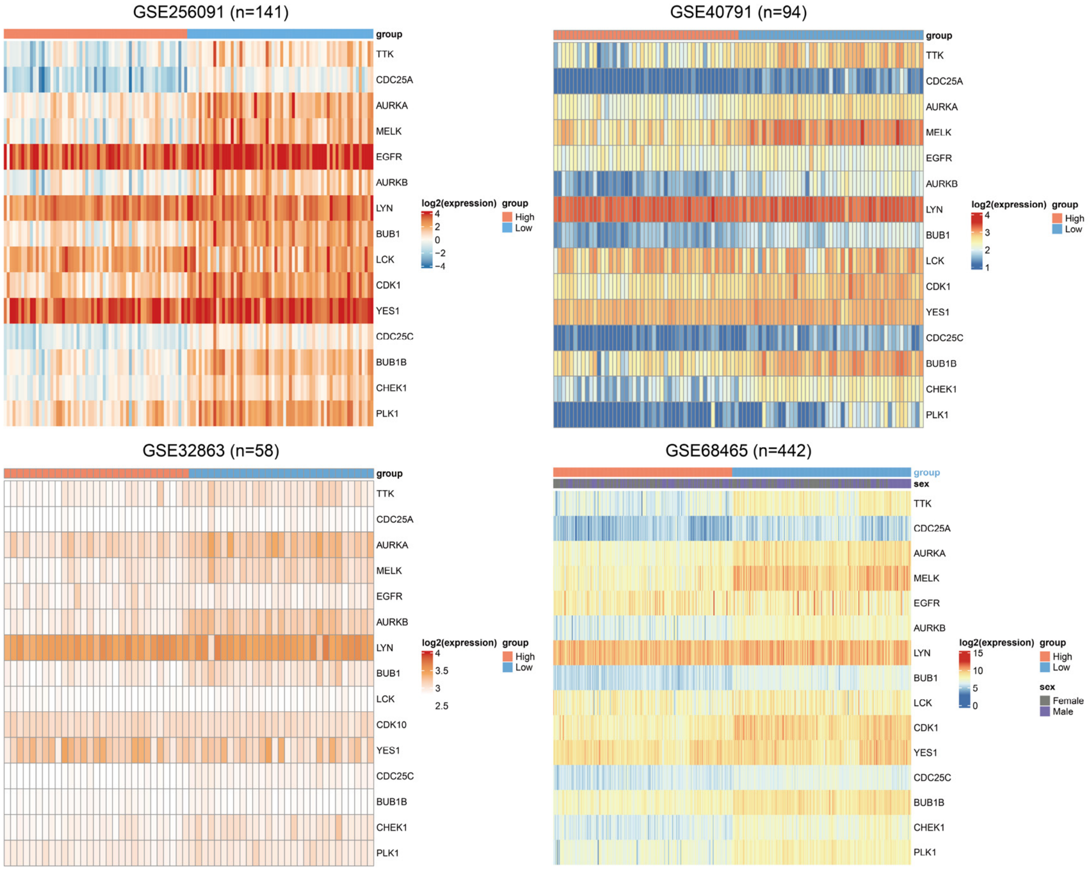

Figure S5. Expression patterns of hub PPMEs between different PSig score groups. n represents the number of LUAD patients in each cohort.

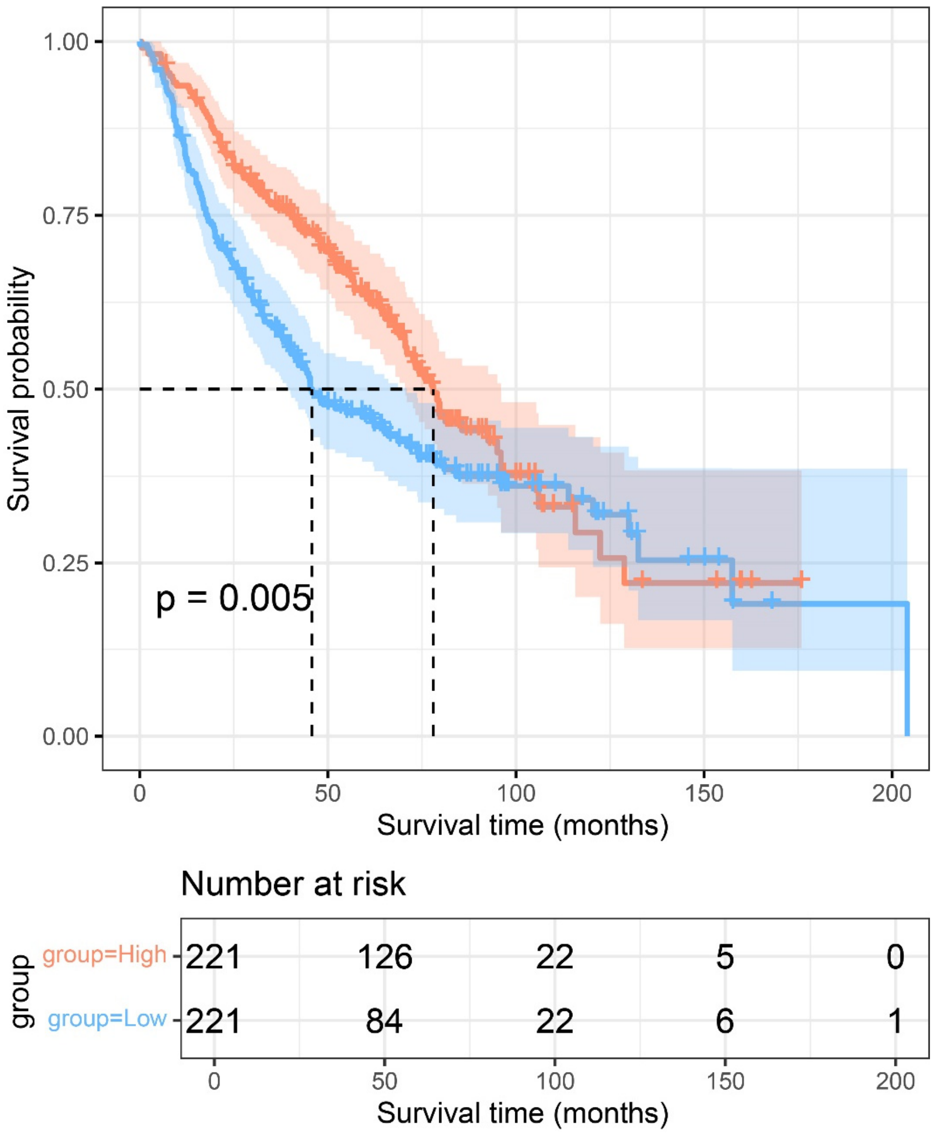

Figure S6. Kaplan-Meier curves showed the difference in survival between the high PSig and low PSig groups in the GSE68465 cohort.
